# Effects of Skin-to-Skin Care on Late Preterm and Term Infants At-Risk for Neonatal Hypoglycemia

**DOI:** 10.1097/pq9.0000000000000030

**Published:** 2017-06-20

**Authors:** Arpitha Chiruvolu, Kimberly K. Miklis, Karen C. Stanzo, Barbara Petrey, Chelsey G. Groves, Kari McCord, Huanying Qin, Sujata Desai, Veeral N. Tolia

**Affiliations:** From the * Department of Women and Infants, Baylor Scott and White Medical Center McKinney and Pediatrix Medical Group, Tex.; † Department of Education and Research, Baylor Scott and White Medical Center McKinney, Tex.; ‡ Department of Quantitative Sciences, Baylor Scott and White Health Care System, Dallas, Tex.; and § Department of Pediatrics, Baylor University Medical Center and Pediatrix Medical Group, Tex.

## Abstract

**Objective:**

The objective of this study was to evaluate the effects of prolonged skin-to-skin care (SSC) during blood glucose monitoring (12–24 hours) in late preterm and term infants at-risk for neonatal hypoglycemia (NH).

**Study design:**

We conducted a retrospective pre- and postintervention study. We compared late preterm and term infants at-risk for NH born in a 1-year period before the SSC intervention, May 1, 2013, to April 30, 2014 (pre-SSC) to at-risk infants born in the year following the implementation of SSC intervention, May 1, 2014, to April 30, 2015 (post-SSC).

**Results:**

The number of hypoglycemia admissions to neonatal intensive care unit among at-risk infants for NH decreased significantly from 8.1% pre-SSC period to 3.5% post-SSC period (*P* = 0.018). The number of infants receiving intravenous dextrose bolus in the newborn nursery also decreased significantly from 5.9% to 2.1% (*P* = 0.02). Number of infants discharged exclusively breastfeeding increased from 36.4% to 45.7%, although not statistically significant (*P* = 0.074).

**Conclusion:**

This SSC intervention, as implemented in our hospital, was associated with a significant decrease in newborn hypoglycemia admissions to neonatal intensive care unit. The SSC intervention was safe and feasible with no adverse events.

## INTRODUCTION

Neonatal hypoglycemia (NH) is a common disorder affecting 5% to 15% of otherwise healthy late preterm and term infants admitted to the newborn nursery.^1^ Infants at-risk for NH include those born late preterm or term infants who are small for gestational age (SGA) or large for gestational age (LGA) or those born to mothers with diabetes.^2^ NH can be defined as the disturbance of normal glucose homeostasis, such that the concentration of glucose in the blood or plasma does not provide adequate delivery of glucose to a target organ (e.g., brain).^3^ Because some cases of prolonged and severe NH may be associated with brain injury and cognitive delay, early identification and management is critical.^4–8^ This often requires administration of formula, which can interrupt breastfeeding or intravenous (IV) dextrose, which may result in mother infant separation, potentially impairing normal metabolic adaptation.^9,10^

Multiple studies have demonstrated that early skin-to-skin contact with the mother improves physiologic stability, including temperature, heart rate, respirations, and blood glucose, particularly in premature and low-birth weight infants.^11–14^ However, no prior study has reported the effect of prolonged skin-to-skin contact on the functional outcomes of NH, such as neonatal intensive care unit (NICU) admissions in otherwise healthy at-risk late preterm and term infants. We hypothesized that prolonged skin-to-skin care (SSC) for at least 12 hours during blood glucose monitoring would significantly decrease NICU admissions for hypoglycemia in infants at risk for NH. In May of 2014, we instituted new guidelines in our mother-infant unit to provide this SSC intervention targeted to otherwise healthy late preterm and term infants at-risk for NH.

## METHODS

### Study Design

We conducted a retrospective pre- and postintervention study utilizing the maternal and neonatal electronic medical records. We compared the late preterm and term infants at-risk for NH born in a 1-year period before the implementation of the SSC intervention, May 1, 2013, to April 30, 2014 (pre-SSC) to the at-risk infants born in the year following the implementation of the SSC intervention, May 1, 2014, to April 30, 2015 (post-SSC). The Institutional Review Board at the Baylor Research Institute (Dallas, Tex.) approved this study. This article was written to conform to the Strengthening the Reporting of Observational studies in Epidemiology guidelines for reporting of cohort studies.^15^

### Cohort Identification and Intervention

All the study infants were born at Baylor Scott and White Medical Center McKinney, a community hospital averaging 1,800 deliveries per year. Infants were deemed at-risk for NH if they were born to mothers with diabetes, SGA, LGA, or born late preterm (35–36 6/7 weeks gestation by best obstetrical dating). Infants were excluded if they had other primary medical reason, such as respiratory distress, sepsis, or neonatal abstinence syndrome that mandated NICU admission. Infants born 34 6/7 weeks gestation or less were also excluded as they were directly admitted to NICU per hospital policy.

**Fig. 1.**
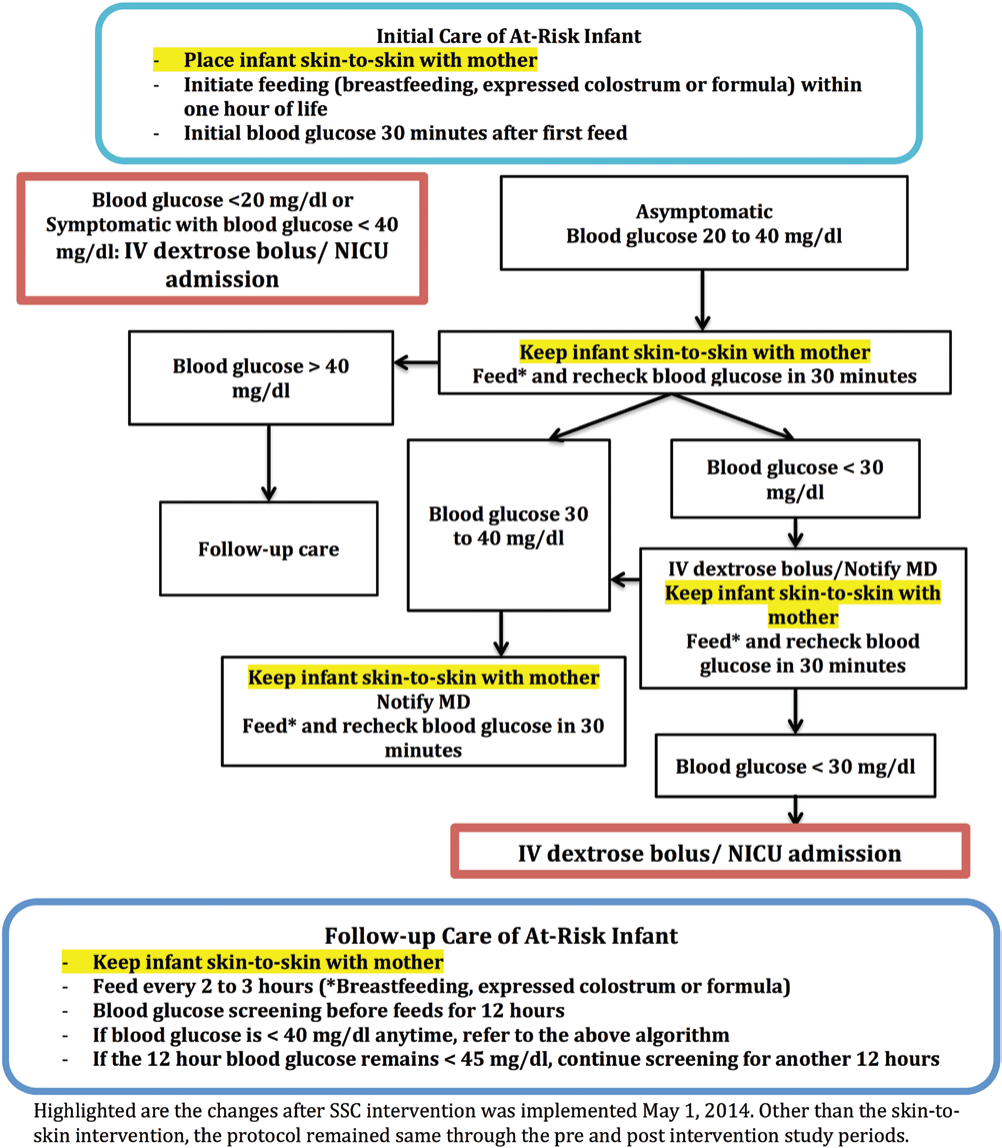
Highlighted are the changes after SSC intervention was implemented May 1, 2014. Other than the skin-to-skin intervention, the protocol remained same through the pre- and postintervention study periods.

Before implementation of the SSC intervention, there was no formal policy on the SSC in the late preterm and term infants at-risk for NH. A standardized postnatal glucose homeostasis (PGH) protocol was followed with blood glucose monitoring, feeding (breastfeeding, expressed colostrum, or formula), IV dextrose bolus, and NICU admission as indicated in Figure 1. Infants at-risk for NH were admitted to NICU anytime if symptomatic or the blood glucose was < 20 mg/dl or remained < 30 mg/dl, despite receiving 1 IV dextrose bolus in the mother-infant unit.

The SSC intervention, implemented May 1, 2014, recommended that all the newly born infants at-risk for NH were to be placed prone on the mother’s bare chest immediately after birth in all vaginal and uncomplicated Cesarean deliveries and dried/covered with a warm blanket across the back. This intervention lasted until blood glucose monitoring was complete according to the PGH protocol. For most infants, the time period of the SSC intervention was 12 hours, out of which the first 2 hours was uninterrupted. However, if the 12-hour blood glucose was < 45 mg/dl, the blood glucose monitoring and SSC intervention were continued for an additional 12-hour period. Infant blood glucose samples were performed before each feed and collected by heel prick while the infant was skin-to-skin with the mother and analyzed immediately with the Precision-Xceed Pro (Abbott Diabetes Care, Alameda, Calif.) glucometer, using compatible blood glucose strips. The nursing staff discussed the importance of this intervention with parents either before or soon after delivery and provided continuous encouragement to the mother during blood glucose sampling for successful implementation. Systematic recording of total time period of intervention was not performed, as we could not place video cameras in the mother-infant unit rooms due to privacy issues. Aside from the SSC intervention, there were no other changes to the PGH protocol that was followed before the intervention (Fig. 1). Donor breast milk was not used in the mother-infant unit.

### Data Collection and Analyses

All the variables that could describe the pre- and postintervention groups and influence the incidence of NH were collected. Maternal data collected included demographics, obstetric complications, labor, and delivery variables. Neonatal data included gestational age, birth weight, gender, and Apgar scores. The proportion of infants born to mothers with diabetes or born late preterm was recorded. The diagnosis of maternal diabetes was categorized from obstetric records either as type 1 or type 2 (diagnosed before pregnancy) or gestational diabetes (diagnosed during pregnancy by oral glucose tolerance testing). Late prematurity was defined as 35–36 6/7 weeks gestation by best obstetric estimate. The proportion of infants born SGA or LGA was also recorded. SGA was defined as < 10 percentile for gestational age and LGA was defined as > 90 percentile for gestational age based on gender-specific intrauterine growth curves by Olsen et al.^16^

Our primary outcome measure was the proportion of at-risk infants with hypoglycemia requiring admission to NICU based on PGH protocol. Secondary outcomes included the proportion of at-risk infants discharged exclusively breastfeeding (defined as infants who were exclusively fed their own mother’s breast milk, with no formula exposure during their hospital stay), need for IV dextrose bolus in the mother-infant unit, and any occurrence of seizures or coma, 2 of the most serious complications of NH.

**Fig. 2.**
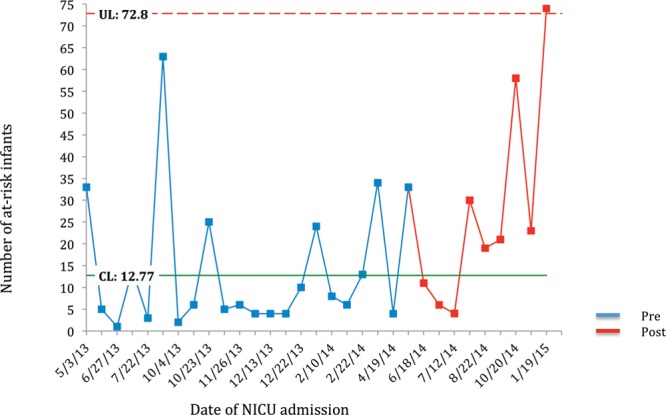
G-chart is developed for infants at-risk for hypoglycemia born and admitted to the mother-infant unit between the NICU admissions. The number of at-risk infants in between admissions increased postintervention. The last 6 points in the chart above the median (CL, Center Line) suggests an improvement postintervention.

**Fig. 3.**
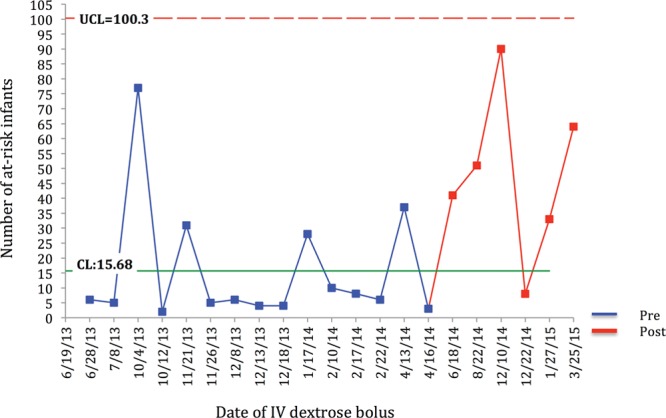
G-chart is developed for infants at-risk for hypoglycemia born and admitted to the mother-infant unit between the IV dextrose boluses. The number of at-risk infants in between bolus increased postintervention. The last 5 of 6 points in the chart above the median (CL) suggest an improvement postintervention.

**Fig. 4.**
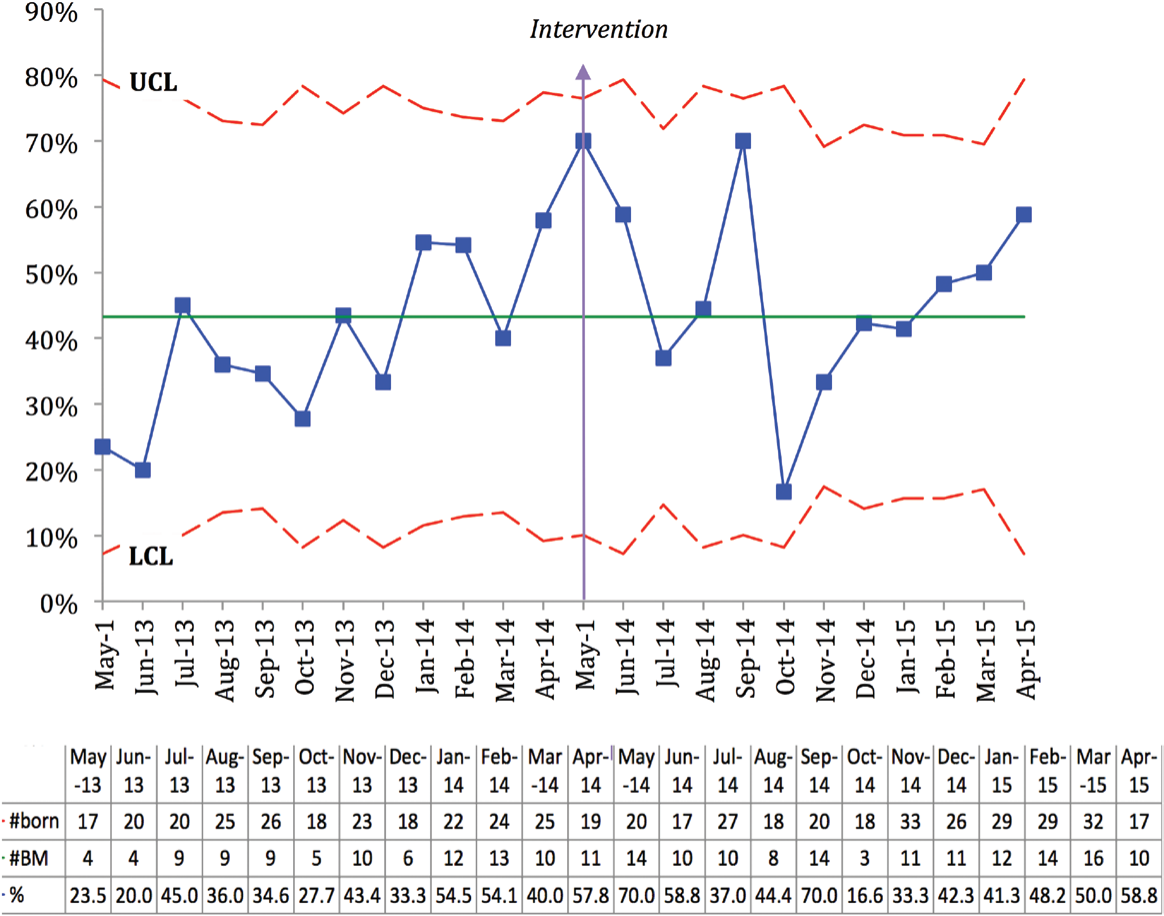
P-chart demonstrates the percentage of infants at-risk for hypoglycemia plotted each month discharged exclusively breastfeeding. BM, exclusive breastfeeding.

All statistical analyses were performed utilizing SAS Enterprise Guide software version 6.1 (SAS Institute Inc., Cary, N.C.). Demographic and outcome variables were compared between the pre-SSC and post-SSC groups with the use of Student’s *t* test for continuous variables and χ^2^ test for categorical variables. A probability value < 0.05 was considered to be the threshold of statistical significance. Time-series analyses utilizing statistical process control methodology was also performed for primary and secondary outcomes including G-charts of at-risk infants born and admitted to the mother-infant unit between NICU admissions (Fig. 2) and IV dextrose boluses administered in the newborn nursery (Fig. 3) and a P-chart of percentage of at-risk infants discharged exclusively breastfeeding (Fig. 4).

## RESULTS

The total number of infants born in the hospital during pre-SSC and post-SSC period were 1,790 and 1,900, respectively. During the pre-SSC period, there were 272 (15.2%) at-risk infants for NH compared with 289 (15.2%) during post-SSC period. These infants were admitted to the mother-infant unit with no other criteria that would mandate NICU admission.

Table 1 compares the maternal and infant characteristics during each time period. There were no significant differences with regard to maternal age, mode of delivery, incidence of multiple gestation, or other obstetric complications, such as pregnancy-induced hypertension, preeclampsia, polyhydramnios, oligohydramnios, rupture of membranes > 18 hours, or chorioamnionitis. Among infant data, there were no significant differences in gestational age, birth weight, gender, or Apgar scores at 1 or 5 minutes. Similarly, the proportion of infants of diabetic mothers, SGA, LGA, or late preterm infants was similar in the 2 time periods.

**Table 1.**
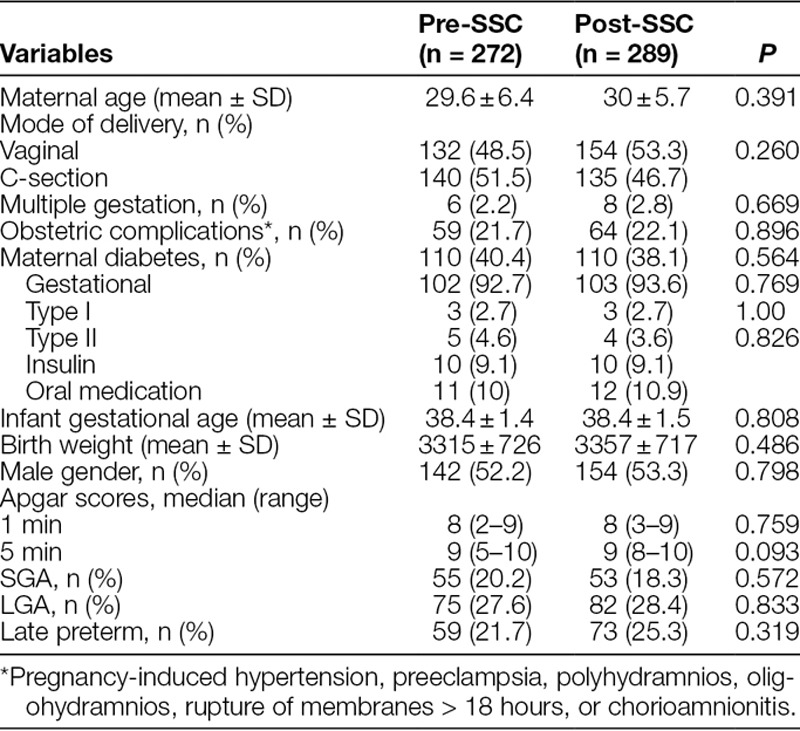
Maternal and Infant Characteristics

Table 2 describes the outcome measures of infants in both periods. The number of hypoglycemia admissions to NICU among at-risk infants for NH decreased significantly from 22 (8.1%) in the pre-SSC period to 10 (3.5%) in the post-SSC period (*P* = 0.018). The number of infants receiving an IV dextrose bolus in the mother-infant unit also decreased significantly from 16 (5.9%) to 6 (2.1%; *P* = 0.02). The percentage of infants exclusively breastfeeding at discharge increased from 36.4% to 45.7%, although this did not meet the level of statistical significance (*P* = 0.07). There were no adverse events associated with the SSC intervention. There were no reports in either group of seizures or coma.

**Table 2.**
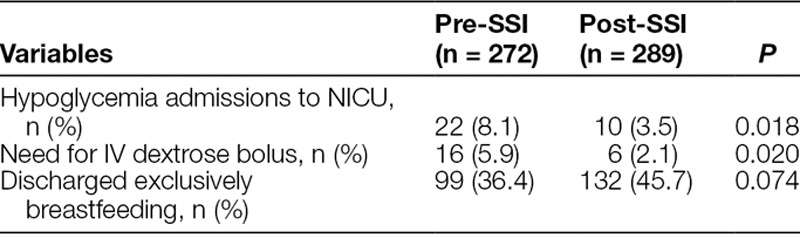
Outcome Measures

G-charts developed for infants born and admitted to the mother-infant unit between the NICU admissions (Fig. 2) and IV dextrose boluses (Fig. 3) demonstrate increased number between events suggesting improvement postintervention. The P-chart demonstrating the percentage of at-risk infants discharged exclusively breastfeeding is also included (Fig. 4).

## DISCUSSION

In this cohort of late preterm and term infants at-risk for NH, the implementation of the SSC intervention decreased both the frequency of NICU admission for hypoglycemia and the use of IV dextrose bolus in the mother-infant unit. Importantly, these reductions were achieved without any negative impact on the rate of exclusive breastfeeding at discharge, which is a Joint Commission perinatal core measure.^17^ Also, there were no adverse events associated with prolonged SSC.^18^

NICU admission can negatively impact both the mother and the infant. The mother may develop stress, anxiety, and depression while the infant is at increased risk for adverse neurodevelopmental outcomes due to early separation from the mother.^19–21^ Colostrum production and breastfeeding may also be adversely affected.^11,14^ The treatments for hypoglycemia, including formula supplementation and IV dextrose may suppress the normal metabolic adaptation in these at-risk infants.^9,10^

The results of this study are consistent with previous reports in which SSC has been reported to be protective against hypoglycemia.^22^ The mechanism of this effect is not well characterized. There is strong evidence that SSC helps with temperature regulation and brown fat conservation in infants.^23^ This may result in stabilization of blood glucose by preventing depletion of glycogen stores. Also, there is evidence that SSC decreases infant neurosteroid levels, which may indicate decreased stress after birth and therefore better energy conservation and blood glucose stabilization.^24^ Several studies have shown that SSC improves colostrum production and the likelihood of exclusive breastfeeding at hospital discharge.^25,26^ Colostrum has been shown to stabilize newborn glucose levels as effectively as formula in the first few hours after birth.^27^ The effect of SSC on infant blood glucose levels may also be due to better transition and improvement in the overall newborn physiologic stability.^14^

This pre- and post-intervention study has several limitations. This study reports observational data of a quality improvement process. Thus, conclusions from this study are limited to associations. It is also possible that results may be biased by other clinical practice changes, although our hospital PGH policy and criteria for NICU admission remained the same during the entire study period. The observed effects of the SSC intervention may also be confounded with other components of SSC, such as breastfeeding. In addition, we were not able to measure the precise length of time that SSC occurred in each at-risk infant as rigorous collection of this data was beyond the scope of this quality improvement project. However, the nursing “charting by exception” did acknowledge general adoption of the intervention during the study period. In a recent meta-analysis, variation in duration did not appear to have an important impact on the effect of the skin-to-skin intervention.^14^ It is also possible that our data were confounded by the Hawthorne effect. Despite these inherent limitations, we also respectfully note that testing this hypothesis with a standardized trial design would be difficult, as there is no equipoise for randomizing mother-infant dyads away from SSC. This study also has limited generalizability because we reported data from a single center. However, we believe that our observation is important as it more likely reflects the real world clinical practice and supports physiologic metabolic adaptation in these at-risk infants. Recent studies have demonstrated the benefits of managing NH with dextrose gel.^28,29^ The implementation of the SSC intervention in this cohort reduced NH interventions to a comparable degree. Still, the SSC intervention and dextrose gel are not mutually exclusive therapies, and there is a need for further studies that combine these 2 approaches.

In conclusion, we successfully implemented the SSC intervention in our hospital in the late preterm and term infants at-risk for NH. This intervention was safe, feasible, and was associated with a decrease in hypoglycemia admissions to NICU and IV dextrose administration in the newborn nursery among at-risk infants for NH. Further clinical studies are necessary to determine if this intervention has other short- or long-term clinical benefits in these infants. SSC may be considered as an intervention in all the newborns, including those not at risk for NH, to prevent early transient newborn hypoglycemia, which has been reported to have long-term implications.^30^ Future studies should include the long-term neurodevelopmental outcomes associated with NH.

## DISCLOSURE

The authors have no financial interest to declare in relation to the content of this article.
